# The Analysis of Bifenox and Dichlobenil Toxicity in Selected Microorganisms and Human Cancer Cells

**DOI:** 10.3390/ijerph16214137

**Published:** 2019-10-27

**Authors:** Agata Jabłońska-Trypuć, Urszula Wydro, Lluis Serra-Majem, Elżbieta Wołejko, Andrzej Butarewicz

**Affiliations:** 1 Division of Chemistry, Biology and Biotechnology, Faculty of Civil Engineering and Environmental Engineering, Bialystok University of Technology, 15-351 Białystok, Poland; u.wydro@pb.edu.pl (U.W.); e.wolejko@pb.edu.pl (E.W.); a.butarewicz@pb.edu.pl (A.B.); 2 Research Institute of Biomedical and Health Sciences, University of Las Palmas de Gran Canaria, 35001 Las Palmas de Gran Canaria, Spain; lserra@dcc.ulpgc.es

**Keywords:** herbicides, bacteria, fungi, ZR-75-1 cells

## Abstract

Bifenox and Dichlobenil belong to the commonly used in Poland in agriculture group of herbicides and their residues are often detected in the environment. They are poorly known regarding their possible carcinogenic and antibacterial effect at the cellular level. Therefore, we decided to study their activity in bacterial strains *Aliivibrio fisheri,* *E. coli, P. aeruginosa,* and *C. albicans* (yeast) and human cancer ZR-75-1 cells. Compounds under study exhibit stimulatory effect on analyzed bacterial strains. The study performed on mammalian cells better reflects the influence of environmental pollutants on human organism, therefore we evaluated the effect of herbicides on ZR-75-1 cells. Cells viability, apoptosis and selected oxidative stress parameters in ZR-75-1 cells were investigated. Both analyzed substances exhibit stimulatory effects on analyzed parameters, however they do not stimulate apoptosis which correlate positively with the induction of oxidative stress. Bifenox and Dichlobenil enhance oxidative stress parameters by the generation of high levels of ROS, which can lead to their adaptation and resistance to the standard treatment regimen.

## 1. Introduction

Exposure to many chemical substances both naturally occurring and of anthropogenic origin may influence the initiation and/or progression of tumors in animals and humans and on the other hand it affects human microbiota [1]. An example of widely distributed environmental contaminants are pesticides, because of their frequent use in agriculture. Selected pesticides and their residues present in human food might increase cancer risk [2,3,4]. Likewise, theirinfluence on microorganisms creating gut microbiota system is incontestable. It was shown that microorganisms play an important role in the metabolism of pesticides, e.g., *Pseudomonas* spp. and *E. coli* are capable of utilizing 3,5,6-trichloro-2-pyridinol as a source of carbon and energy [5]. Therefore, it was concluded that the exposure to pesticides significantly affect the composition and function of human microbiota [6]. 

One of the commonly used groups of pesticides are herbicides design specifically for the control of unwanted vegetation and therefore frequently applied in forestry, agriculture, pasture systems and urban green areas. Literature data indicate possibility of their transport and contamination of surface waters, which increases the risk of environmental contamination [7,8]. Subsequently pollution by herbicides may cause dangerous effects in organisms that are important elements of the food chain, such as primary producers, which are the basis of the food web for aquatic organisms [9]. 

According to IUPAC (International Union of Pure and Applied Chemistry) Bifenox chemical name is methyl 5-(2,4-dichlorophenoxy)-2-nitrobenzoate and it belongs to the group of nitrophenyl ether herbicides. Bifenox belongs to the group of selective herbicides and is used to control annual broad-leaved weeds and selected grasses in many crops, e.g., cereals, maize, soybean, rice and many others. Its action at the cellular level consists in damage to cell membranes and inhibition of photosynthesis. In order to broaden the spectrum of activity, it is often used in combination with other herbicides [10]. It is commonly detected in aquatic environment at the concentration levels which are considered toxic for aquatic organisms [11]. However, there is scarce of data on the effects of Bifenox on the human body at the cellular level, in particular its potential carcinogenic activity and its impact on human microbiome.

The herbicide 2,6-dichlorobenzonitrile (DCBN) named Dichlobenil is a member of the benzonitrile herbicides group. It has been widely used in agriculture and households to control growth of weeds and its residues, mainly its metabolic product 2,6-dichlorobenzamide (BAM) generated by microorganisms, persist in the environment. Therefore, in the year 2008 it was prohibited as an herbicide by the European Union [12]. The mechanism of Dichlobenil toxic effects to target organisms was described as the inhibition of plant cell wall biosynthesis [13]. The toxicity of Dichlobenil was analyzed in many different prokaryotic and eukaryotic systems and an information regarding its acute and chronic toxicity, carcinogenicity, and mutagenicity are available [14,15]. However, there is a significant lack of information available for the toxicity, mutagenicity, carcinogenicity and mechanisms of action of Dichlobenil in human breast cancer cells.

This paper aims to evaluate the degree of hazard of selected herbicides application. Two compounds of this herbicidal group, Bifenox and Dichlobenil, are currently approved for commercial use in the European Union, including Poland. Therefore, it seems interesting to study their influence on human and bacterial cells. In vitro testes which are usually cell- or bacteria-based bioassays represent an excellent screening tool appropriate for high sample throughput. Biological experimental models are indispensable for detecting unknown properties of selected chemical compounds and in comparison to other bioassays especially human cell-based assays have the highest sensitivity for the detection of environmental toxicants.

## 2. Materials and Methods

### 2.1. Reagents

RPMI-1640 medium, containing glucose at 4.5 mg/mL (25 mM), penicillin, streptomycin, trypsin–EDTA, FBS and PBS (without Ca and Mg, pH 7.4) were provided by PAN Biotech. GSH/GSSG-Glo™ Assay kit, Caspase 3/7 Assay and Caspase 9 Assay were provided by Promega Madison, WI. SDS, TCA, TBA, Folin-Ciocalteu reagent were provided by Sigma-Aldrich and DTNB by Serva. Dichlorodihydrofluorescein diacetate assay (DCFH-DA). Bifenox and Dichlobenil and Mueller Hinton II Broth were provided by Sigma-Aldrich, St. Louis, MO, USA. Cell stain double staining kit containing propionium iodide and calcein—AM was provided by Sigma-Aldrich, St. Louis, MO, USA. MTT reagent (3-(4,5-dimethylthiazol-2-yl)-2,5-diphenyltetrazolium bromide) was purchased from Sigma-Aldrich and fluorescein isothiocyanate (FITC) Annexin V Apoptosis Detection Kit I from BD Pharmingen (San Diego, CA, USA). 

### 2.2. Cell Culture

The effect of Bifenox and Dichlobenil was examined in ZR-75-1 breast cancer cell line, which were obtained from American Type Culture Collection (ATCC). Cells were maintained in RPMI-1640 supplemented with 10% FBS, penicillin (100 U/mL), and streptomycin (100 μg/mL) at 37 °C in a humified atmosphere of 5% CO_2_ in air. Adherent cells (2 × 10^4^ cells/mL) in 200 µL of culture medium were incubated with or without the test compounds in tissue culture white and black 96-well plates for fluorescence and luminescence measurements. Apoptosis, GSH/GSSG ratio, ROS level were examined at 0.025 µM and 0.01 µM concentrations of Bifenox and 0.1 µM and 0.05 µM concentration of Dichlobenil. The incubation time was 24 h. For the estimation of TBARS and SH groups content cells were seeded into 6-well plates in 2 mL of culture medium with and without test compounds at above mentioned concentrations. Concentrations for the experiments: 0.025 µM and 0.01 µM concentrations of Bifenox and 0.1 µM and 0.05 µM concentration of Dichlobenil, were chosen on the basis of previously obtained results already presented in the literature [16].

### 2.3. Bacterial Strains

*E. coli* (ATCC 25922), *P. aeruginosa* (ATCC27853) and *C. albicans* (ATCC 10231) were obtained from the American Type Culture Collection (Manassas, VA, USA). Selected strains are model strains of bacteria and fungi, respectively which are used in antimicrobial tests. *E. coli, P. aeruginosa* (Gram negative bacteria) and *C. albicans* (fungi) were grown overnight in Mueller Hinton II Broth at 37 °C (bacterial strains) and 27 °C (fungi). Next day, the overnight cultures were diluted in fresh MH II Broth to obtain 10^8^ CFU/mL (CFU – colony forming units) for bacteria and 10^6^ CFU/mL for fungi. For the antimicrobial activity was used the inoculum where the suspension of *E. coli* and *P. aeruginosa* cells was 10^6^ CFU/mL and *C. albicans* was 10^4^ CFU/mL.

### 2.4. Chemical Treatment of Cells

Bifenox and Dichlobenil were stored in a refrigerator at temperature 4 °C. 

Bacterial cells were treated with increasing concentrations of Bifenox and Dichlobenil in the range from 0.01 µM to 200 µM.

The compounds were added to the cultured human cells for a final concentrations: 0.025 µM and 0.01 µM of Bifenox and 0.1 µM and 0.05 µM of Dichlobenil. The control cells were incubated without the test compounds.

### 2.5. Microtox Toxicity Test

The ecotoxicity analysis was carried out in accordance with the PN-EN ISO 11348-3: 2008 standard [17]. The *Aliivibrio fischeri* luminescent bacteria, previously known as *Vibrio fischeri* [18], were exposed to two types of pesticides: Dichlobenil and Bifenox in above mentioned range of concentrations. *Aliivibrio fischerii* emits light as a result of natural metabolic processes taking place in cells after the contact with the analyzed sample, causing changes in the intensity of light emission by these organisms [19]. The luminescence measurement was carried out using Microtox^®^ Model 500 analyzer (Strategic Diagnostics Inc., Newark, NJ, USA), based on the manufacturer’s procedure—“81.9% Basic Test”. Before performing the Microtox Basic Test^®^, the samples were appropriately prepared for the assays. Sodium chloride solution was used to adjust the osmotic pression of the samples to approximately 2%. Toxicity assessments were made after 5 and 15 min. Omni 4.1 supporting computer software with a standard log-linear model was used to calculate the effective concentration values (EC_50_) of the tested samples. In order to determine the toxicity of investigated samples the calculated values of the EC_50_ (after 15 min.) were converted into units of acute toxicity (*TUa*) according to the procedure specified for liquid phase calculation:

*TU_a_* = 100/*LC*_50_ (1)

The obtained *TU_a_* values of the liquid phase were classified into specific toxicity classes according to the to the toxicity classification system (TCS) proposed by Persoone and are presented in Table 1 [20].

## 3. Method of Determining Antibacterial Activity Assay

Antimicrobial activity of tested pesticides against of *E. coli*, *P. aeruginosa* and *C. albicans* was evaluated based on MTT colorimetric assay (MCA) [21,22]. An aliquote of 90 µL of the indicator strains suspension incubated with tested herbicides for 24, 48 and 72 h at 37 °C were transferred to a sterile 96 well plate. Next, 10 µL of 5 mg/mL MTT solution was added into each well and incubated for 4 h. After that, the cultures were centrifuged at 1000× *g* for 10 min. to precipitate formazan crystals and the supernatant was removed. Then 100 µL of acid isopropanol (0.1 M HCl in isopropanol) was added and the mixture was incubated and shaken for 10 min. at room temperature. The optical density (OD) of the formazan solution was measured at the wavelength of 560 nm using a microplate reader GloMax^®^-Multi Microplate Multimode Reader. 

The antimicrobial activity of Bifenox and Dichlobenil was presented as a degree of the reduction or stimulation in bacterial/fungal cells viability after 24, 48 and 72 h in compared to the control cells (without tested herbicides) and expressed as a percentage (%). All experiments/assays/tests were run in triplicates.

### 3.1. Estimation of Tested Compounds Cytotoxicity

Cytotoxicity tests were conducted previously and the results were described in literature [16]. On the basis of obtained results the most stimulatory concentrations were chosen for the experiments. For Bifenox the most stimulatory concentration were 0.025 µM and 0.01 µM and for Dichlobenil −0.1 µM and 0.05 µM.

### 3.2. Determination of Caspase 3/7 Activity

ZR-75-1 cells were seeded in 96-well white plates at a density 2 × 10^4^ cells/well. Cells were cultured for 24 h and were treated with Bifenox and Dichlobenil in a concentrations 0.025 µM and 0.01 µM of Bifenox and 0.1 µM and 0.05 µM of Dichlobenil. After intended time of incubation medium was removed and Assay reagents were added to the wells. The activity of caspases 3/7 was measured by using luminescent assay based on the substrate which contains the tetrapeptide sequence DEVD in a reagent optimized for caspase activity, luciferase activity and cell lysis. Cell lysis is followed by the caspase cleavage of the substrate and generation of the luminescent signal. Luminescence is proportional to the amount of caspase activity present in the sample. Assay is based on the thermostable luciferase activity, which generates stable, “glow-type” luminescent signal. The luminescence was measured in a microplate plate reader GloMax^®^-Multi Microplate Multimode Reader. All the experiments were done in triplicates. 

### 3.3. Determination of Caspase 9 Activity

ZR-75-1 cells were seeded in 96-well white plates at a density 2 × 10^4^ cells/well. Cells were cultured for 24 h and 48 h and were treated with Bifenox and Dichlobenil in a concentrations 0.025 µM and 0.01 µM of Bifenox and 0.1 µM and 0.05 µM of Dichlobenil. After intended time of incubation medium was removed and Assay reagents were added to the wells. The activity of caspase 9 was measured by using luminescent assay based on a luminogenic caspase-9 substrate in a buffer system provided for caspase activity, luciferase activity and cell lysis. The addition of a special Caspase-Glo^®^ 9 Reagent results in cell lysis, followed by caspase cleavage of the substrate, and generation of a “glow-type” luminescent signal. The signal generated is proportional to the amount of caspase activity present in the sample. The assay is based on thermostable luciferase, which generates the stable “glow-type” luminescent signal. The luminescence was measured in a microplate plate reader GloMax^®^-Multi Microplate Multimode Reader. All the experiments were done in triplicates. 

### 3.4. Flow Cytometry Analysis of Apoptosis

ZR-75-1 cells were exposed to 0.01 µM B, 0.025 µM B, 0.05 µM D and 0.1 µM D and incubated for 24 h. Apoptosis was evaluated by flow cytometry on FACSCanto II cytometer (Becton-Dickinson). The cells were trypsinized and resuspended in DMEM and RPMI-1640. After that time, the cells were suspended in binding buffer for staining with FITC-Annexin V and propidium iodide—PI for 15 min at room temperature in the dark following the manufacturer’s instructions (FITC Annexin V apoptosis detection Kit I). The signal obtained from cells stained with Annexin V or PI alone was used for fluorescence compensation. Data were analyzed with FACSDiva software (BD PharmingenTM, San Diego, CA, USA). 

### 3.5. Total Protein Content in Cells

Adherent cells (2.5 × 10^5^ cells/mL) in 2 mL of culture medium were incubated with or without the test compounds in tissue culture 6-well plates. After the homogenization of ZR-75-1 cells and extraction in 0.1 M NaOH at 4 °C total protein content was calculated. The concentration of proteins was determined spectrophotometrically as per Lowry. Folin phenol reagent with a protein kit calibrated with bovine serum albumin as the standard was used in the experiment. The absorbance of the extracts was measured spectrophotometrically at 750 nm [23]. All the experiments were done in triplicates. 

### 3.6. Determination of TBA Reactive Species (TBARS) Levels

The level of TBA-reactive species (TBARS) as membrane lipid peroxidation markers was measured using the method of Rice-Evans et al. as described previously [23]. Adherent cells (2.5 × 10^5^ cells/mL) in 2 mL of culture medium were incubated with or without the test compounds in tissue culture 6-well plates. The cells were washed with PBS (pH 7.4), scraped from Petri dishes and subsequently resuspended in 1 mL of PBS (between 1 and 5 × 10^6^/plate). TCA (15%, 1 mL) and TBA (0.37%, 1 mL) were added to 1 mL of the cell suspension and mixed. This mixture was submerged in a boiling water bath for 10 min and the concentration of TBARS was assessed spectrophotometrically at 532 nm using the extinction coefficient of 156 mM/cm. All the experiments were done in triplicates. 

### 3.7. Determination of SH Groups

SH-groups were measured using the method of Rice-Evans (1991) as described previously [23]. Adherent cells (2.5 × 10^5^ cells/mL) in 2 mL of culture medium were incubated with or without the test compounds in tissue culture 6-well plates. Cells were washed twice with PBS (pH = 7.4; 4 °C) and dispersed by scraping. MCF-7 cells were counted, resuspended in 1 mL of PBS and collected by centrifugation at 5000× *g* for 10 min. The pellet was resuspended in 1 mL of 0.5 M phosphate buffer (pH 7.8), containing 0.1% SDS. Subsequently, 25 μL Ellman’s reagent (5 mM) was added and the thiol groups were measured spectrophotometrically at 412 nm using the molar extinction coefficient of 13.6 mM-1 cm-1. All the experiments were done in triplicates.

### 3.8. Determination of GSH/GSSG

Total glutathione and GSH/GSSG ratio were each assayed in triplicate via GSH/GSSG-Glo™ kit (Promega Corporation, Madison, WI, USA) following manufacturer’s instructions. Cells were seeded in white bottom 96-well plates at 10^4^ cells/well (Sarstedt), allowed to attach, and treated with pesticides. Prior to the assay growth media were removed and cells washed with PBS. Assay is based on a luminescence measurement and detect and quantify total glutathione (GSH + GSSG), GSSG and GSH/GSSG ratios in cultured cells. Stable luminescent signals are correlated with either the GSH or GSSG concentration of a sample. In this method GSH-dependent conversion of a GSH probe, Luciferin-NT, to luciferin by a glutathione S-transferase enzyme is coupled to a firefly luciferase reaction. Light from luciferase depends on the amount of luciferin formed, which in turn depends on the amount of GSH present. Thus, the luminescent signal is proportional to the amount of GSH. GSH/GSSG ratios are calculated directly from luminescence measurements. Luminescence was read using the GloMax^®^-Multi Detection System. All the experiments were done in triplicates.

### 3.9. Intracellular ROS Detection

The level of intracellular reactive oxygen species (ROS) was determined using dichlorodihydrofluorescein diacetate (DCFH-DA), (Sigma, St. Louis, MO, USA) [24]. After diffusion through the cell membrane, DCFH-DA is deacetylated by cellular esterases to a non-fluorescent compound, which is later oxidized by intracellular ROS into a fluorescent 2′, 7′–dichlorofluorescein (DCF). The ZR-75-1 cells (2 × 10^4^) were seeded in 200 µL of growth medium in black 96-well plates. After 24 h, the medium was removed, the cells were stained with 10 μM of DCFH-DA in PBS at 37 °C, 5% CO_2_ incubator, for 45 min. Next, the dye was removed and replaced with pesticides in 0.025 µM and 0.01 µM of Bifenox and 0.1 µM and 0.05 µM of Dichlobenil concentrations and incubated for 24 h. Then, the DCF fluorescence intensity was measured by using the GloMax^®^-Multi Detection System at the excitation wavelength of 485 nm and the emission wavelength of 535 nm. The intracellular ROS generation in pesticides-stimulated ZR-75-1 cells was shown as the intensity of fluorescence of the DCF. All the experiments were done in triplicates. 

### 3.10. Fluorescent Microscopy Assay

For apoptotic and necrotic cells nuclear morphology evaluation fluorescent dyes, such as propidium iodide and calcein-AM were used. The ZR-75-1 cells were seeded on cell imaging dishes with coverglass bottom with 0.025 µM and 0.01 µM concentrations of Bifenox and 0.1 µM and 0.05 µM concentrations of Dichlobenil and without tested compound as a control, for 24 h. After incubation the cells were washed twice with PBS and then stained with dyes solution in the dark in 37 °C, for 15 min. After incubation, staining mixture was removed and the cells were washed with PBS and analyzed by using fluorescent microscope (200× magnification). Calcein-AM stains only viable cells and PI can only pass through disordered areas of dead cell membrane. The cells were analyzed and photographed via using Olympus IX83 fluorescent microscope with SC180 camera with Cell Sens Dimension 1.17 program. The following criteria were used: living cells - have regularly distributed green chromatin nucleus and are stained with green color and dead cells, probably apoptotic cells are characterized by red nuclei with chromatin condensation or fragmentation, while necrotic cells showed red-stained cell nuclei. 

### 3.11. Statistical Analysis

All data are given as mean values ±SEM (standard error of means). Differences between treatments and untreated control cells were analyzed by one-way ANOVA, followed by Dunnett’s procedure for multiple comparisons. Significant effects are represented by *p* ≤ 0.05 (*), *p* ≤ 0.01 (**), *p* ≤ 0.001 (***). For parametric data one-way analysis of variance (ANOVA) followed Tuckey test was applied. Results were expressed as mean ± standard deviation (SD) of mean for parametric data. Significance was considered when *p* ≤ 0.05. The relationships among different variables determined by PCA (Principal Components Analysis) which is based on Pearson’s correlation and was presents as biplot graph. The analysis allows to visualize the variation present in a dataset with many variables. Statistica 13.0 (StatSoft, Kraków, Poland) was used. 

## 4. Results

### 4.1. Toxicity Tests 

The results of performed toxicity tests of pesticides samples are presented in Table 2. Based on the calculated acute toxicity units of the tested pesticide samples, the appropriate toxicity classes were determined.

According to obtained results presented in Table 2, from the two analyzed pesticides, Bifenox was characterized by the significantly higher toxicity. This is confirmed by the test results presented in the safety data sheet for the test agent in which it obtained category 1 in relation to the aquatic environment. This pesticide is very toxic to aquatic life with long-term effects.

So far, however, the mutagenic effect of this agent on reproductive cells has not been determined. There are no data on the persistence and degradability, bioaccumulation and mobility of Bifenox in soil. Similarly, there is no such data for Dichlobenil. At the same time, in the safety data sheet of this pesticide, the 4 toxicity category was determined. Concentrations lower than 50 µM were not toxic in case of both analyzed compounds.

### 4.2. Antibacterial Activity of Analyzed Herbicides

**Figure 1:**
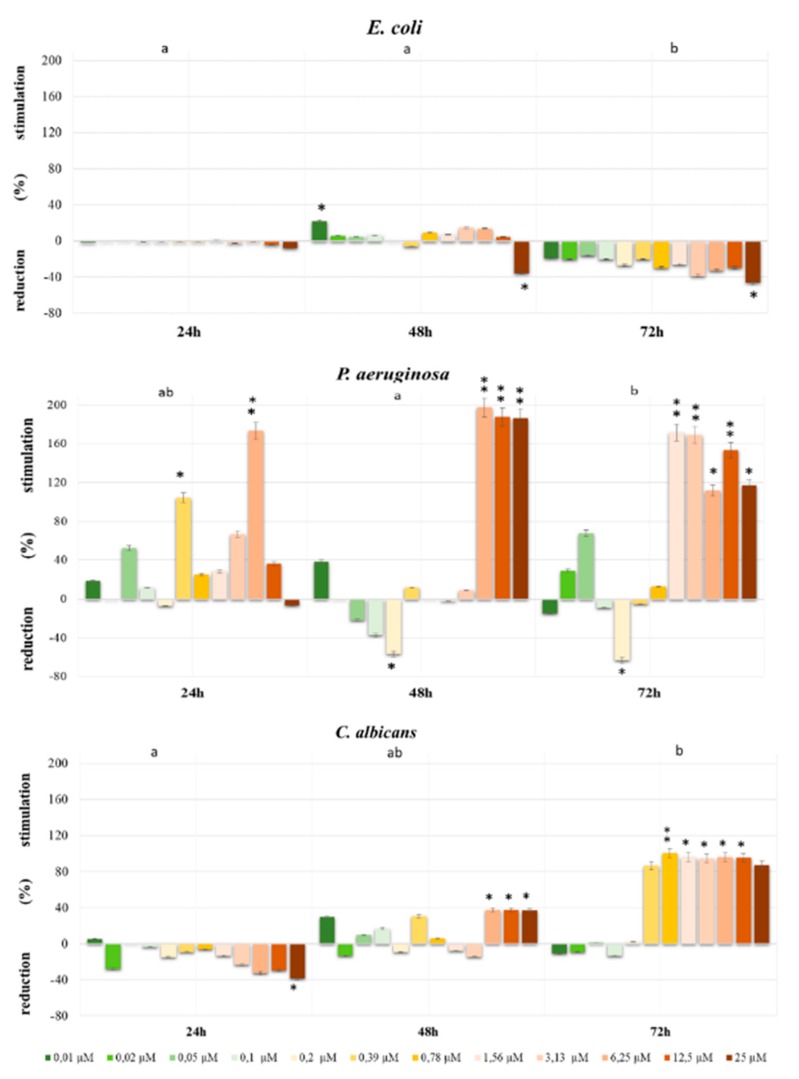
The growth stimulation/reduction of *E. coli, P. aeruginosa* and *C. albicans* exposed to Bifenox for 24 h, 48 h and 72 h calculated as a percentage of control. The different letters indicate significant statistical differences (*p* ≤ 0.05) between each time of incubation estimated by Tukey’s test. *—*p* < 0.05, **—*p* < 0.01, ***—*p* < 0.001 represent significant effects between treatments and control were assessed by Dunnett test.

**Figure 2:**
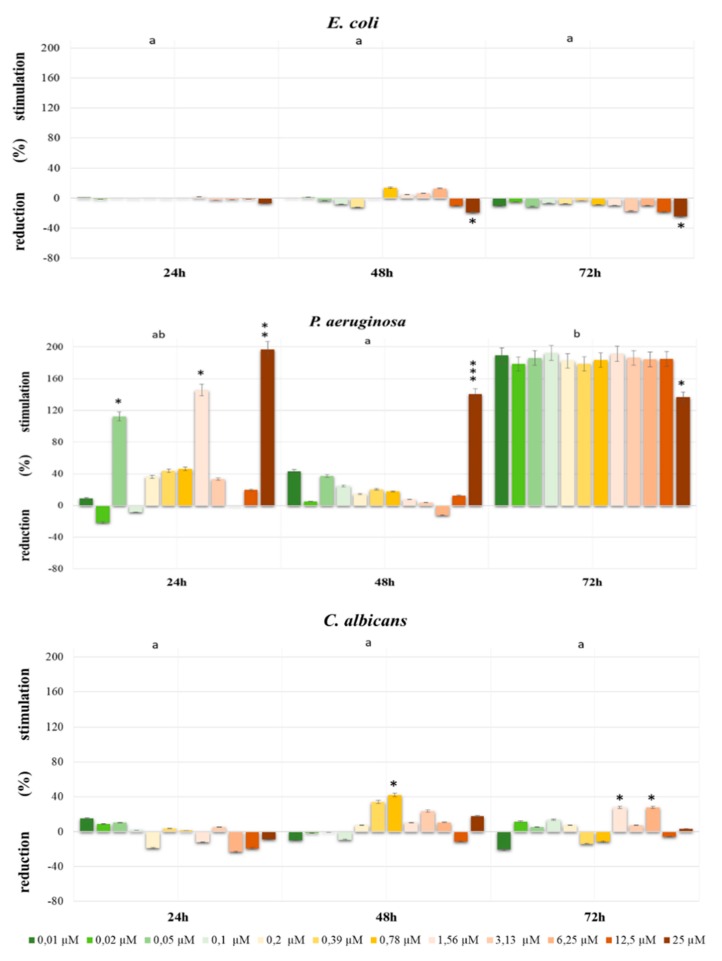
The growth stimulation/reduction of *E. coli, P. aeruginosa* and *C. albicans* exposed to Dichlobenil for 24 h, 48 h and 72 h calculated as a percentage of control. The different letters indicate significant statistical differences (*p* ≤ 0.05) between each time of incubation estimated by Tukey’s test. *—*p* < 0.05, **—*p* < 0.01, ***—*p* < 0.001 represent significant effects between treatments and control were assessed by Dunnett test.

The obtained results showed different effects after the application of the herbicides for *E. coli*, *P. aeruginosa* and *C. albicans* strains (Figure 1 and Figure 2). After 48 h for *E. coli* the significant stimulating effect (22%, *p* < 0.05) was observed at the concentration of 0.1 µM Bifenox. The significant reduction of its effect for *E. coli* growth was found after 48 h and 72 h (35% and 46%, respectively) in a dose of 25 µM (*p* < 0.05). The most significant stimulating effect of Bifenox for *P. aeruginosa* growth was observed after 48 h in doses of 6.25 µM; 12.5 µM; 25 µM, at *p* < 0.01 and after 72 h in doses of 1.56 µM; 3.13 µM; 12.5 µM, at *p* < 0.01 and 6.25 µM; 25 µM, at *p* < 0.05. In turn, for *C. albicans* the most significant increase in cell viability under the influence of Bifenox was found after 48 h in doses of 6.25 µM, 12.5 µM, 25 µM, at *p* < 0.05 and after 72 h in doses of 0.78 µM, at *p* < 0.01 and 1.56 µM, 3.13 µM, 6.25 µM, 12.5 µM, at *p* < 0.05 (Figure 1).

Figure 2 indicates the effect of Dichlobenil on *P. aeruginosa* growth after 72 h in all doses used in the experiment, while after 24 h and 48 h the most significant stimulating effect in the concentration of 25 µM Dichlobenil was observed. The reduction effect of Dichlobenil for *E. coli* growth was found after 24 h and 72 h in all doses used in the experiment. However, the statistically significant increase in *C. albicans* viability after 48 h in doses of 0.78 µM, at *p* < 0.05 and after 72 h in doses of 1.56 µM and 3.13 µM, at *p* < 0.05 was observed (Figure 2). 

### 4.3. Caspases Activity

**Figure 3:**
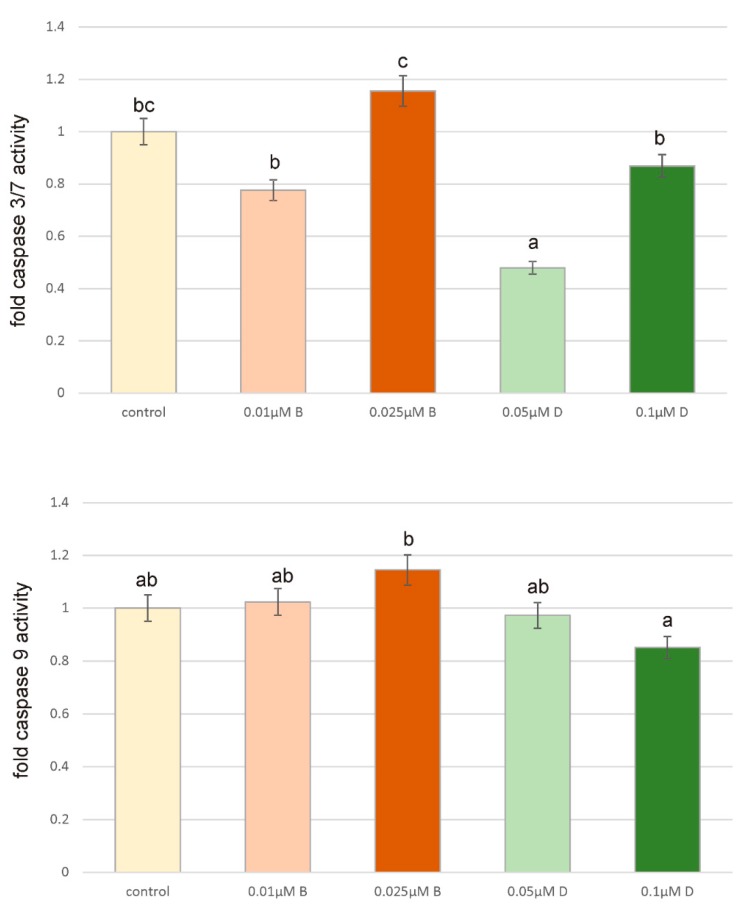
The effect of Bifenox (B) and Dichlobenil (D) on caspase 3/7 and caspase 9 activity in ZR-75-1 cells. The cells were incubated with 0.01 µM B, 0.025 µM B, 0.05 µM D and 0.1 µM D, for 24 h. Mean values from three independent experiments ± SEM are shown. Different letters indicate statistical differences (*p* ≤ 0.05) between each treatment estimated by Tukey’s test.

The level of caspases activity under the influence of two herbicides in selected concentrations was analyzed as a marker of apoptosis and it is presented in Figure 3. Caspases 3/7 are the primary effector caspases in apoptosis pathway. They can be activated by caspase 9, which trigger apoptosis through proteolysis of anti-apoptotic proteins (ICAD, Bcl-2). Analyzed compounds did not significantly increase the level of caspase 3/7 and caspase 9 activity. The level of caspase 3/7 was higher as compared to control only in case of Bifenox 0.025 µM treatment, but an observed increase was not statistically significant. Similar results were obtained for caspase 9 activity. Only Bifenox in 0.025 µM concentration caused an increase in caspase 9 activity as compared to control. Dichlobenil in both tested concentrations caused decrease in caspases activity. Obtained results indicate that studied pesticides do not stimulate apoptosis in breast cancer cells. 

### 4.4. Detection of Apoptosis by Flow Cytometry

**Figure 4:**
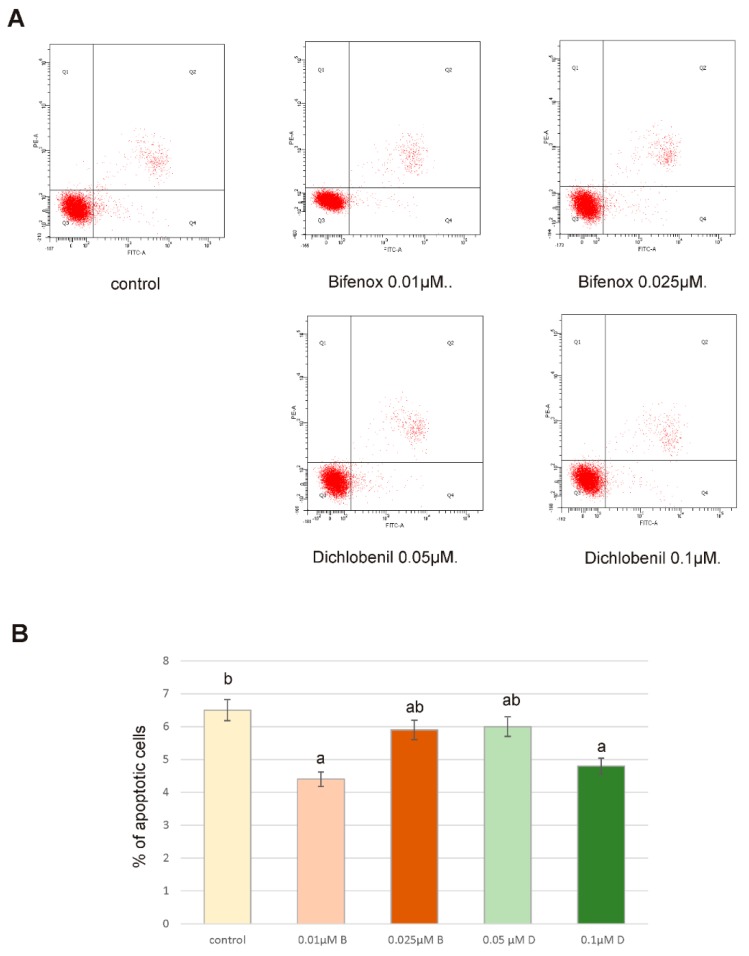
The effect of Bifenox and Dichlobenil on apoptosis of ZR-75-1 cells. The cells were incubated with 0.01 µM B, 0.025 µM B, 0.05 µM D and 0.1 µM D, for 24 h. Representative dot plotes for 24 h treatment cells subjected to Annexin V-FITC/propidium iodide (PI) staining are shown (**A**). Bar graphs presenting the percentage of apoptotic (**B**) cells are demonstrated. Mean values from three independent experiments ± SD are shown. Different letters (a,b) indicate statistical differences (*p* ≤ 0.05) between control, Bifenox treatment and Dichlobenil treatment estimated by Tukey’s test.

Apoptosis was analyzed by flow cytometry on FACSCanto II cytometer (Becton-Dickinson, San Diego, CA, USA). Figure 4 depicts the percent of apoptotic cells in ZR-75-1 culture incubated for 24 h with 0.01 µM Bifenox, 0.025 µM Bifenox, 0.05 µM Dichlobenil and 0.1 µM Dichlobenil. Statistically significant differences between control untreated cells and pesticides treated cells indicate that analyzed compounds did not enhance apoptosis, because all obtained for pesticides values are below control level. Obtained results confirm above described low caspases activity. 

### 4.5. Oxidative Stress Parameters

**Figure 5:**
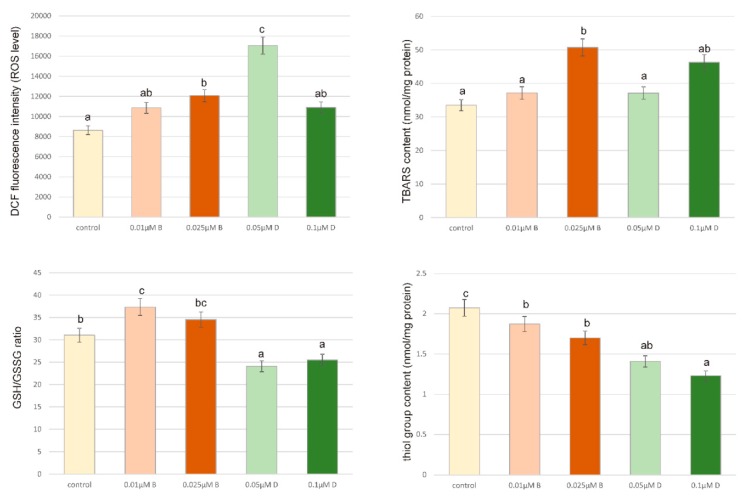
The effect of Bifenox (B) and Dichlobenil (D) on the level of intracellular ROS, GSH/GSSG ratio, TBARS content and SH group content in ZR-75-1 cells. The cells were incubated with 0.01 µM B, 0.025 µM B, 0.05 µM D and 0.1 µM D, for 24 h. Mean values from three independent experiments ± SEM are shown. Different letters indicate statistical differences (*p* ≤ 0.05) between each treatment estimated by Tukey’s test.

Figure 5 indicates the effect of Bifenox and Dichlobenil on ROS generation in ZR-75-1 cells. It presents the fluorescence intensity of 2′7′-dichlorodihydrofluorescein (DCF) for ZR-75-1 cells incubated with selected herbicides for 24 h. Treatment of cells with different concentrations of analyzed pesticides caused an increase in ROS generation. It was particularly observed in case of Dichlobenil in 0.05 µM concentration, which caused almost two-fold increase in ROS concentration as compared to control untreated cells. Lower concentration of herbicide is more efficient in ROS stimulation than higher, but every studied concentration of both compounds is efficient in the stimulation of the oxidative stress.

The study of reduced glutathione content is essential in the estimation of oxidative stress parameters, because it is one of the most important low-molecular mass antioxidant, which plays an important role in maintaining oxidative homeostasis. Figure 5 shows rather stimulatory effect of analyzed pesticides on GSH amount in ZR-75-1 cells. Increases in GSH/GSSG ratio were observed especially under the influence of Bifenox in both tested concentrations. Dichlobenil did not show such a stimulating effect on the content of the tested parameter.

In cancer cells, in contrast to normal healthy cells, lipid peroxidation process serves as an important source of reactive species, which stimulate cancer cell proliferation. As expected, both the tested compounds significantly increased the level of lipid peroxidation, especially in the higher concentrations (Figure 5). The obtained results demonstrate that both Bifenox and Dichlobenil exhibit pro-oxidative properties, stimulate TBARS production and membrane lipid peroxidation. Figure 5 contains also the level of SH-groups for ZR-75-1 cells exposed to Bifenox and Dichlobenil for 24 h at selected concentrations. Presented results show the most significant increase in thiol group content of 68% compared to untreated control cells after 24 h treatment at a Dichlobenil concentration of 0.1 µM. Both tested pesticides increased SH group content in all tested concentrations as compared to the control. Presented results revealed no significant oxidative damage in cellular protein after 24 h treatment under the influence of both Bifenox and Dichlobenil.

### 4.6. Fluorescent Microscopy Assay

**Figure 6:**
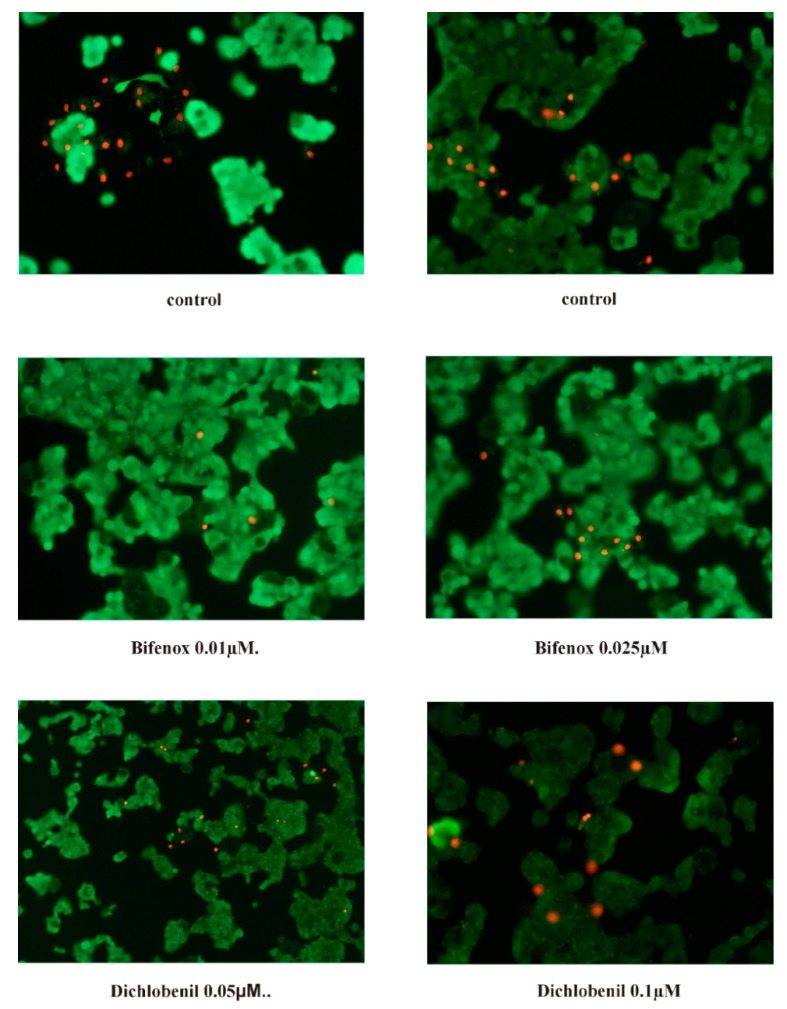
The effect of Bifenox (B) and Dichlobenil (D) on apoptosis and necrosis in the ZR-75-1 cell line evaluated by fluorescence microscope assay (200× magnification). The cells were incubated with 0.01 µM B, 0.025 µM B, 0.05 µM D and0.1 µM D, for 24 h and stained with Calcein-AM and propidium iodide. We present representative images form one of three independent experiments.

For the confirmation of apoptosis process evaluated by above described methods, fluorescent staining was used (Figure 6). Similarly, to luminescent assay we did not observe significant changes in control and pesticides-treated cells. Calcein—AM only stains viable cells and propidium iodide stain viable and death cells, respectively. We did not observe significant changes in cells viability, which confirm results obtained in MTT assay, presented previously in literature data [13]. 

### 4.7. Statistical Analysis of Studied Parameters

**Figure 7:**
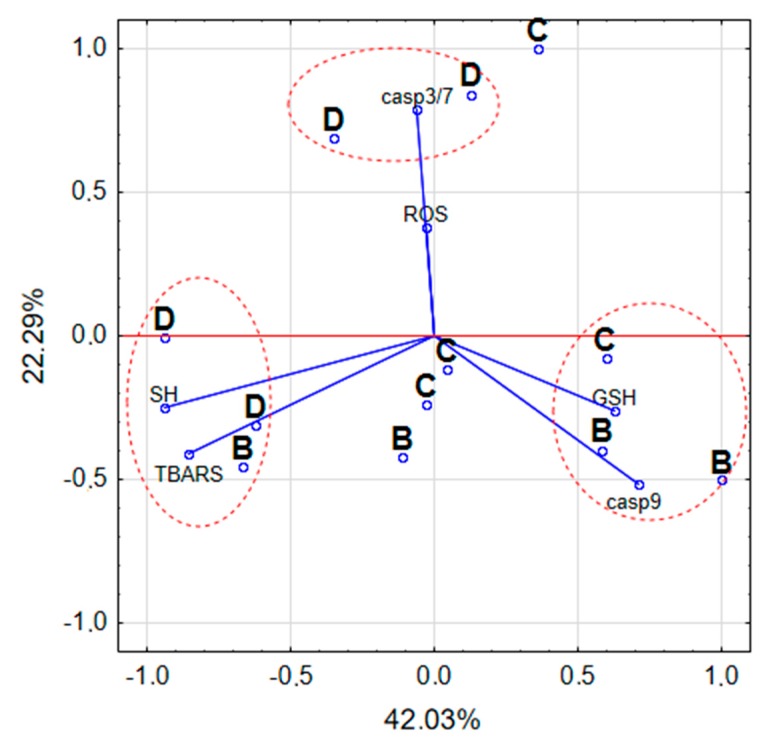
Biplot representing both variables (oxidative stress parameters: SH groups, TBARS content, GSH/GSSG ratio; caspases: caspase 3/7, caspase 9) and cases (tested pesticides: B-Bifenox, D-Dichlobenil, C-control) in two dimensions.

Figure 7 demonstrated PCA analysis which shows the relationship between the study variables regarding the oxidative stress caused by the tested pesticides and caspases activities and GSH content in ZR-75-1 breast cancer cells. As presented in Figure 7, activity of Dichlobenil is positively correlated with the activity of caspase 3/7 and is also correlated with the second component which explained 22% variability. On the other hand, Bifenox activity is especially correlated with caspase 9 activity and GSH/GSSG ratio, which are represented by the first component which explained 42% variability.

## 5. Discussion

Pesticides are widely used in many branches of agriculture to control weeds, insects and plant pathogens and to stimulate plant growth. Crop plants are exposed to pesticides, which can easily get to the human digestive tract through daily meals. Low but long-term exposure to pesticides can cause dangerous effects on the skin, endocrine system, nervous system and many other systems of the human body. The mechanism of the action of pesticides is usually based on the production of free radicals that can cause lipid peroxidation, DNA damage, cell death and possible carcinogenic effects [25].

Although the discussion about the activity of pesticides as carcinogenic pollutants is relevant and according to the literature data majority of utilized pesticides influence also human gut microbiome, very little attention has been paid to possible interactions with autochthonous human microbiota, especially that with pathogenic potential [26,27]. *E. coli* is very popular bacterium model utilized for studying bacterial response mechanisms to selected xenobiotic, e.g., environmental contaminants exposure, therefore, we also estimated the influence of both selected herbicides on it. Our results indicate decreases in *E. coli* cells viability under the influence of both Bifenox and Dichlobenil, which is consistent with literature data presenting that selected herbicides cause an arrest in *E. coli* divisions right after exposure [28]. On the other hand, *P. aeruginosa* is also a gram-negative bacteria, which causes a variety of diseases, from mild cutaneous infections to complicated pulmonary complications [29]. Lima IS et al. indicated that continuous exposure to low doses of selected herbicides significantly stimulated *P. aeruginosa* growth, which is also in line with our results. We observed statistically significant increases in *P. aeruginosa* cells proliferation, especially under the influence of Dichlobenil. Observed increases in the intensity of bacterial cells proliferation can be also explained by the fact that herbicides may serve as a source of carbon, which could be processed in both aerobic and anaerobic conditions and, especially *Pseudomonas* spp., may produce glycine with the use of pesticides, which subsequently enhance their growth [30,31,32]. In case of *C. albicans* initially we observed decreases in cell growth under the influence of both studied compounds, which is in agreement with recent findings indicating that selected acetohydroxyacid synthase (AHAS) inhibitors developed as commercial herbicides may represent a new class of antifungal drug candidates simultaneously exhibiting low toxicity to human cells [33]. However, it should be also mentioned that elsewhere, Wachowska U. claimed that some pesticides may stimulate the growth of *C. albicans*, what we observed after 72 h of treatment [34]. 

An excessive level of oxidative stress causes cancer cell adaptation, an increase in cancer cells proliferation and genetic instability, which leads to strong resistance to anticancer therapy [35]. Although the research has tended to focus on selected pesticides chemical analysis rather less attention has been paid to their mechanisms of action, especially on the cellular level. Therefore, we decided to study Bifenox and Dichlobenil influence on apoptosis and oxidative stress parameters in estrogen-dependent breast cancer cells. The main point of this paper was to reveal if two selected herbicides commonly used in Poland in agriculture area may exhibit stimulatory effect on oxidative stress parameters and subsequently influence cancer cell viability simultaneously inhibiting apoptosis. The results of cytotoxicity studies of two selected herbicides were obtained previously and are presented in literature [16]. On the basis of above mentioned results we chose the most stimulatory concentrations for further analysis. As an experimental model we chose ZR-75-1 breast cancer cell line which is an estrogen-dependent cell line and may be used for endocrine-based study. The concentrations which were chosen for the experiments are environmentally relevant, because compounds from the analyzed group are often found in groundwater system in such concentration range. Rich et al. claimed also that similar concentration range has stimulatory effect on different, estrogen-dependent breast cancer cell lines, which in consistence with our data [36]. Most studies have emphasized on an indisputable connection between oxidative stress and cancer initiation, promotion and progression [37]. Elevated levels of ROS is an extremely important factor in the pathogenesis of breast cancer because it results in the emergence of genetic and epigenetic modification, which then cause uncontrolled cell proliferation [38,39]. Tumor cells usually show elevated levels of ROS compared to normal healthy cells. This is due to the fact that an increased oxidative stress caused by ROS can subsequently resulted in a decrease in the level of antioxidant defense in the body. It is the main line of defense of the body against angiogenesis and cancer metastases, which are the main stages in the development and progression of cancer [40,41]. This is consistent with results obtained in our experiments. We observed an increase in ROS generation in ZR-75-1 cells under the influence of both Bifenox and Dichlobenil as compared to control, untreated cells. In case of 0.1 µM concentration of Dichlobenil a decrease in ROS content was observed as compared to 0.05 µM concentration of Dichlobenil, however still we observed an increase in analyzed parameter comparing to control untreated cells. Such increases usually coexist with increases in cancer cell proliferation, which was also indicated in our study. Obtained results also indicate increases in TBARS content, especially in higher concentrations of both analyzed compounds, which draws primarily on the work of Gonenc et al., 2005, who indicated that the content of lipid peroxidation products increases in various types of cancer, including breast cancer [42]. We also noticed that SH group content in breast cancer cells stimulated with pesticides correlated with TBARS level. Observed decreases in analyzed parameter are in accordance with literature data indicating herbicides pro-oxidative properties against cell membrane proteins [43,44]. It is also in accordance with GSH/GSSG ratio decreases observed especially in case of Dichlobenil in both tested concentrations. Bifenox especially in 0.01 µM concentration caused statistically significant increase in GSH/GSSG ratio, which could be confusing, but it may also be related to the level of SH groups, which is higher under the influence of Bifenox than Dichlobenil. Literature data points out that GSH decreases in breast cancer are being noticed and acts as a biomarker of oxidative stress [45]. 

The presented results allowed us to conclude that oxidative damage of proteins, high level of lipid peroxidation and increase in ROS content may cause changes in signaling pathways and cause the aforementioned increase in the level of ZR-75-1 cancer cell proliferation and their resistance to the process of apoptosis. However, we did not observe any significant changes in caspases activity and therefore apoptosis triggering. Caspase 3/7 are effector caspases in the apoptosis pathway, which can be activated by caspase 9, which subsequently triggers apoptosis through anti-apoptotic proteins proteolysis. Caspase 9 is activated by the binding of cytochrome c, which is released after the mitochondrial damage caused by oxidative stress or other factors [46]. Since tested compounds did not cause any significant effect on caspases activity, we did not observe any apoptotic cells by using microscopy fluorescent assay and results obtained from flow cytometry analysis also confirm lack of apoptotic cells it is possible that under the influence of analyzed herbicides ZR-75-1 cells acquire resistance to apoptosis. Acquired apoptosis resistance is a hallmark of most human cancers and among environmental chemicals that contribute to this situation pesticides are mentioned very often [47]. 

PCA analysis confirms positive correlation of Dichlobenil with caspase 3/7 activity. Obtained results indicate for statistically significant decrease in caspase 3/7 activity under the influence of Dichlobenil, which was presented in Figure 5. Both analyzed compounds are in correlation with SH group content and TBARS, which was also demonstrated by using PCA analysis. An observed changes in TBARS content accompanied SH group content, which relate with increased level of oxidative stress and this phenomenon intensifies cancer cell proliferation. It is in accordance with literature data indicating that oxidative stress plays an important role in the development of breast cancer [41]. Results presented for the activity of caspase 9 and GSH/GSSG ratio under the influence of Bifenox are connected and represented by one component. 

Although a variety of cell lines and bacterial strains are being used for the estimation of toxicity of selected environmental pollutants, one of the most common and most simple approach to assess cytotoxicity is the measurement of bioluminescence inhibition of naturally bioluminescent bacteria—*Aliivibrio fischeri* [48]. Obtained results indicated that studied herbicides were not toxic to this bacterial model strain in concentrations lower than 50 µM. Despite the lack of toxicity of the analyzed substances on the model strain (*Aliivibrio fischeri*), they were found to be potentially dangerous for humans. This is due to the fact that the lower, tested concentrations of herbicides caused significant changes both in the tested strains (bacteria and fungi) and in human cells.

## 6. Conclusions

An extensive body of literature indicate that pesticides induce oxidative stress in both normal healthy cells and cancer cells. The chronic exposure to oxidative stress leads not only to increased cell growth and survival, but also in increased tumorigenic potential of estrogen-dependent breast cancer cells. Obtained results indicate stimulatory influence of Bifenox and Dichlobenil on bacterial and human cancer cells, however further study regarding molecular mechanisms of pesticides action in both presented biological models are needed. 

## Figures and Tables

**Table 1:** Toxicity classification system of environmental samples [20].

Toxicity Class	TUa	Evaluation of the Toxicity of the Sample
0	O	no acute toxicity
I	<1	low acute toxicity
II	1–10	acute toxicity
III	10–100	high acute toxicity
IV	≥100	very high acute toxicity

**Table 2:** Microtox test results of analyzed pesticides samples.

Type of Pesticides	Incubation Time [min]		Toxicity Class
	15		
	EC_50_ [%]	TUa	
Dichlobenil (200 µM)	3.736	26.77	II
Bifenox (200 µM)	51.050	1.96	III
